# Provegetarian Food Pattern and Obesity Among Saudi Non‐Pregnant Females of Reproductive Age

**DOI:** 10.1155/bmri/4023284

**Published:** 2026-09-29

**Authors:** Eman M. Alissa, Samiah A. Algarni, Azza J. Khaffji, Nisma M. Al Mansouri

**Affiliations:** ^1^ Faculty of Medicine, King Abdulaziz University, Jeddah, Saudi Arabia, kau.edu.sa; ^2^ Obstetrics and Gynecology, King Abdulaziz Hospital and Oncology Center, Jeddah, Saudi Arabia

**Keywords:** BMI, diet pattern, FFQ, vegetarian, waist circumference, waist-to-hip ratio

## Abstract

**Aim:**

Evidence linking plant‐based dietary patterns with adiposity in Middle Eastern populations is limited. This study examined associations between provegetarian diet index (PDI) scores and measures of general and central adiposity among Saudi women of reproductive age.

**Methods:**

This cross‐sectional study included 205 non‐pregnant Saudi women aged 18–40 years systematically recruited from two tertiary hospitals in Jeddah. Dietary intake was assessed using a validated 91‐item food frequency questionnaire and used to calculate PDI scores. Anthropometric measures included body mass index (BMI), waist circumference (WC), waist‐to‐hip ratio (WHR) and waist‐to‐height ratio (WHtR). Multivariable regression models were adjusted for potential confounders.

**Results:**

Higher PDI scores were generally characterised by higher intakes of fibre, polyunsaturated fatty acids, vitamin C and *β*‐carotene and lower intakes of saturated fatty acids and cholesterol. PDI score was weakly inversely correlated with WHR (*r* = −0.144, *p* < 0.05). Adjusted analyses showed no significant associations with continuous BMI, WC, WHR or WHtR (all *p* > 0.05). Higher PDI scores were associated with modestly lower odds of WHR‐defined central obesity (adjusted OR = 0.972; 95% CI: 0.947–0.997; *p* = 0.031), whereas associations with WC‐ and WHtR‐defined central obesity were not significant.

**Conclusion:**

PDI scores were not consistently associated with measures of general or central adiposity. Although higher scores were associated with modestly lower odds of WHR‐defined central obesity, this isolated finding was not supported by the continuous adiposity outcomes and should be considered exploratory. Prospective studies are needed to determine whether this association is reproducible.

## 1. Introduction

Plant‐based diets have gained increasing global attention because of their potential benefits for weight management and cardiometabolic health [1]. Obesity is a major cardiometabolic risk factor and an important public health concern in Saudi Arabia, where approximately 20% of the population is affected, with a particularly high burden reported among women [2]. Dietary patterns characterized by a greater consumption of plant foods and a lower consumption of animal foods, particularly red and processed meats, have been proposed as potentially beneficial for maintaining a healthier body weight and adiposity profile [3–5].

Plant‐based dietary patterns vary considerably in the extent to which they restrict animal‐derived foods, ranging from strict vegan and vegetarian diets to more flexible approaches [6]. The provegetarian, or flexitarian, dietary pattern emphasizes greater consumption of plant‐based foods while allowing lower or less frequent consumption of animal‐derived foods, including meat, fish, eggs and dairy products [7]. Rather than requiring complete exclusion of animal foods, the provegetarian approach therefore reflects the relative preference for plant foods within the overall diet.

The relationship between dietary patterns and adiposity may differ according to the anthropometric measure considered. Body mass index (BMI) is widely used to characterize general adiposity, whereas measures such as waist circumference (WC) and waist‐to‐hip ratio (WHR) provide complementary information on central fat distribution [8, 9]. Examining both general and central adiposity may therefore provide a more comprehensive understanding of the relationship between provegetarian dietary adherence and obesity‐related phenotypes. This is particularly relevant among women of reproductive age, given the important metabolic and reproductive health implications of obesity [10].

Because foods and nutrients are consumed in combination, assessment of overall dietary patterns may better capture their cumulative and potentially interactive relationships with adiposity than consideration of individual dietary components alone. The provegetarian diet index developed by Martínez‐González et al. [11] provides a quantitative measure of the relative preference for plant‐derived over animal‐derived foods without requiring adherence to a strict vegetarian diet. However, evidence regarding the relationship between the provegetarian diet index and adiposity remains limited, particularly among women in Saudi Arabia. Furthermore, little is known about whether adherence to this dietary pattern shows consistent associations across measures of general and central adiposity in this population

Therefore, the present study is aimed at examining the association between adherence to the provegetarian diet index and measures of general and central adiposity among Saudi non‐pregnant females of reproductive age

## 2. Methods

A total of 205 non‐pregnant females of reproductive age, ranging from 18 to 40 years old, attending the obstetrics and gynaecology clinics at King Abdulaziz University Hospital and the King Abdulaziz Hospital and Oncology Center in Jeddah, Saudi Arabia, for routine preventive care and health assessment, were randomly selected to participate in this cross‐sectional study. Participants were recruited using systematic random sampling. Following a randomly selected starting point, every fifth eligible woman attending the clinics for routine preventive care was approached for participation until the required sample size was achieved. Every participant provided their signature on the informed consent document, and the study project received approval from the study ethics committees of both hospitals in accordance with the ethical standards outlined in the Declaration of Helsinki. This study is reported in accordance with the Strengthening the Reporting of Observational Studies in Epidemiology (STROBE)(Available here) statement for observational research.

The exclusion criteria encompass females aged over 40, who have chronic conditions such as cardiovascular diseases, inflammatory disorders such as rheumatoid arthritis, thyroid diseases, infections, the use of nonsteroidal anti‐inflammatory medicines, hormonal contraceptive use, corticosteroid therapy, micronutrient supplements, or who are pregnant.

Data about sociodemographic characteristics, lifestyle behaviours, health problems and medication usage were collected using a standardised questionnaire administered in a face‐to‐face interview. The assessment of physical activity level was conducted using the validated Arabic version of the International Physical Activity Questionnaire [12], and the results were expressed in MET‐min/week.

The final score of the provegetarian diet index was determined by evaluating the dietary information using a validated semi‐quantitative food frequency questionnaire (FFQ) consisting of 91 items [13]. The FFQ gathered data on the usual dietary consumption for the preceding year. Each item had a set average portion size, and consumption frequencies were recorded using five categories, ranging from ‘*nearly never*’ to ‘*2–4 times/day*’. The daily intake (in grammes per day) was determined by multiplying the recommended portion size of each food item, as outlined in the Saudi Dietary Guidelines, by the frequency of consumption [14]. The nutritional content of foods was assessed using the McCance and Widdowson’s The Composition of Food tables [15], with supplementary information [16]. Individuals having unrealistic numbers for the average daily energy intake (less than 500 or greater than 3500 kcal/day) were not included in the current study.

The provegetarian diet index was calculated according to the scoring approach described by Martínez‐González et al. [11]. The index comprised 12 food groups: 7 plant‐derived food groups (fruits, vegetables, nuts, cereals, legumes, olive oil and potatoes) and 5 animal‐derived food groups (animal fats, eggs, fish, dairy products and meats and meat products). The consumption (grammes/day) of all dietary categories was corrected for total energy intake using the residuals approach [17]. Energy‐adjusted intakes of each food group were ranked into quintiles. For plant‐derived food groups, participants in the lowest to highest intake quintiles received scores of 1 to 5, respectively. Animal‐derived food groups were reverse scored, such that participants in the lowest to highest intake quintiles received scores of 5 to 1, respectively. The 12 component scores were summed for each participant to generate the total provegetarian diet index, with higher total scores indicating a greater relative preference for plant‐derived over animal‐derived foods. The final results for the provegetarian diet index varied from 12 (indicating the lowest adherence) to 60 (indicating the maximum adherence). The adherence to provegetarian diet index was classified into 5 categories using the cut‐offs proposed by Martínez‐González et al.: *very low* (≤ 29 points), *low* (30–34 points), *moderate* (35–39 points), *high* (40–44 points) and *very high* (≥ 45 points) [11].

The weight, height, WC and hip circumference were assessed by trained nurses using a calibrated scale, a wall‐mounted stadiometer and a non‐elastic tape. The measurements were taken with the participants wearing light clothing and no shoes (Seca Corp., located in Columbia, Maryland). BMI was computed by dividing the weight (in kilogrammes) by the height (in metres squared). The women were categorised into different BMI statuses based on the World Health Organization categorisation [9]. These categories include underweight, normal weight, overweight and obese, with corresponding BMI ranges of < 18.5, 18.5–24.9, 25–29.9 and ≥ 30.0 kg/m^2^, respectively. The WC was measured at the midpoint between the lowest rib and the iliac crest. The hip circumference was assessed at its maximum width. WHR and waist‐to‐height ratio (WHtR) were computed for all participants in the research. The study used many criteria to determine central obesity in the participants. These criteria included a WC of 80 cm or more for women, WHR of ≥ 0.85 in women and WHtR > 0.55 in women [18, 19]. Blood pressure readings were obtained using a mercury sphygmomanometer three times, and the average of the three measures was recorded.

The data were reported as the mean ± standard deviation (SD) for numerical data that followed a normal distribution, or as the median (interquartile range (IQR)) for numerical data that did not follow a normal distribution. Categorical variables were provided as numbers (percentages). The normal distribution of all variables was assessed using the Kolmogorov–Smirnov test. For categorical analyses, participants were classified according to their total provegetarian diet index scores into five mutually exclusive score categories. These categories were used for descriptive and subgroup analyses and were not sample‐based quintiles of the total provegetarian diet index. The study utilised one‐way analysis of variance (ANOVA) for normally distributed data and Kruskal–Wallis H test for data that were not normally distributed to identify significant differences across the provegetarian diet index categories. The chi‐square test was employed to compare categorical variables. Pearson or Spearman′s correlation analysis was used to investigate the relationship between the continuous provegetarian diet index and explanatory factors, as deemed suitable. Separate multiple linear regression models were performed with BMI, WC, WHR and WHtR as dependent variables and the continuous provegetarian diet index as the main independent variable. To further examine the association between continuous provegetarian diet index and central obesity, multivariable binary logistic regression models were performed using central obesity defined according to WC (> 80 cm), WHR (≥ 0.85) and WHtR (> 0.55) as binary dependent variables. Models were adjusted for age, marital status, parity, occupation, monthly income, physical activity level, presence of PCOS and total energy intake as potential confounders selected a priori based on their biological and epidemiological relevance. The same covariates were included in the logistic regression models. Linear regression results are presented as unstandardized regression coefficients (B) with 95% confidence intervals (CIs), whereas logistic regression results are presented as odds ratios (ORs) with 95% CIs. All *p* values are two‐tailed, and statistical significance was determined using the standard threshold *p* < 0.05. The statistical analyses were conducted using IBM SPSS Statistics for Windows, Version 21 (IBM Corp., Armonk, New York, USA).

## 3. Results

The observed provegetarian diet index scores in the present study ranged from 16 to 48. A total of 205 females were classified into 5 groups according to provegetarian diet index score categories (≤ 29, 30–34, 35–39, 40–44 and ≥ 45), comprising 18, 62, 75, 40 and 10 participants, respectively. As demonstrated in Table 1, no statistically significant between‐category differences were detected in age, sociodemographic variables, blood pressure readings and anthropometric measurements (*p* > 0.05). Notably, over half of the study individuals were either overweight or obese.

**Table 1:** Clinical characteristics of the study population as stratified by adherence to provegetarian diet index categories (*N* = 205).

	Provegetarian diet index					*p*
	Category 1	Category 2	Category 3	Category 4	Category 5	
	≤ 29 (*very low*)	30–34 (*low*)	35–39 (*moderate*)	40‐44 (*high*)	≥ 45 (*very high*)	
Number	18	62	75	40	10	—
Age (years)	28.7 ± 5.1	29.6 ± 5.9	30.1 ± 5.5	29.3 ± 5.6	29.0 ± 6.6	NS
SBP (mmHg)	118.2 ± 9.5	120.1 ± 9.7	119.8 ± 7.6	120.9 ± 6.3	115.5 ± 9.9	NS
DBP (mmHg)	73.5 ± 8.9	73.6 ± 8.4	73.1 ± 9.9	76.6 ± 9.2	67.4 ± 6.1	NS
Weight (kg)	69.5 ± 14.5	66.3 ± 15.4	68.9 ± 17.7	69.7 ± 15.8	69.6 ± 13.6	NS
Height (cm)	157.1 ± 4.4	156.7 ± 6.9	156.3 ± 5.8	158.3 ± 5.9	156.2 ± 4.2	NS
BMI (kg/m^2^)	28.3 ± 6.9	26.9 ± 5.7	28.1 ± 6.8	27.8 ± 6.1	28.5 ± 5.6	NS
WC (cm)	90.7 ± 10.9	89.4 ± 13.4	90.1 ± 13.8	90.8 ± 13.7	91.0 ± 17.7	NS
HC (cm)	111.2 ± 11.9	109.2 ± 12.5	109.9 ± 12.9	109.9 ± 11.8	110.1 ± 13.8	NS
WHR	0.816 ± 0.06	0.817 ± 0.06	0.818 ± 0.06	0.824 ± 0.07	0.822 ± 0.09	NS
WHtR	0.58 ± 0.1	0.57 ± 0.1	0.58 ± 0.1	0.57 ± 0.0	0.58 ± 0.1	NS
Marital status						
Single	2 (11)	16 (26)	14 (19)	10 (25)	1 (10)	NS
Married	16 (89)	46 (74)	61 (81)	29 (73)	8 (80)	
Divorced	0 (0)	0 (0)	0 (0)	1 (3)	1 (10)	
Parity						
0	9 (50)	38 (61)	44 (59)	25 (63)	4 (40)	NS
1	4 (22)	8 (13)	12 (16)	6 (15)	2 (20)	
2	4 (22)	6 (10)	12 (16)	4 (10)	4 (40)	
≥ 3	1 (6)	10 (16)	7 (9)	5 (13)	0 (0)	
Education level						
Elementary school or less	4 (22)	9 (15)	12 (16)	12 (30)	4 (40)	NS
High school	2 (11)	19 (31)	23 (31)	6 (15)	2 (20)	
Bachelor degree	12 (67)	31 (50)	37 (49)	17 (43)	3 (30)	
Graduate degree	0 (0)	3 (5)	3 (4)	5 (13)	1 (10)	
Occupation						
Student	3 (17)	11 (18)	12 (16)	5 (13)	1 (10)	NS
Housewife	7 (39)	27 (44)	38 (51)	20 (50)	7 (70)	
Office worker	6 (33)	13 (21)	10 (13)	8 (20)	0 (0)	
Managerial post	2 (11)	11 (18)	15 (20)	7 (18)	2 (20)	
Monthly income						
< 5000 SR	1 (6)	16 (26)	14 (19)	11 (28)	4 (40)	NS
5000–10,000 SR	13 (72)	28 (45)	44 (59)	16 (40)	5 (50)	
10,000–20,000 SR	4 (22)	15 (24)	12 (16)	12 (30)	1 (10)	
> 20,000 SR	0 (0)	3 (5)	5 (7)	1 (3)	0 (0)	
Physical activity level (MET‐min/week)	561.7 ± 823.8	438.9 ± 527.0	782.4 ± 1181.4	708.0 ± 1181.4	1503.0 ± 2896.4	NS
Smoking habits						
Non‐smoker	15 (83)	54 (87)	60 (80)	38 (95)	8 (80)	NS
Ex‐smoker	1 (6)	1 (2)	2 (3)	1 (2.5)	0 (0)	
Current smoker	2 (11)	7 (11)	13 (17)	1 (2.5)	2 (20)	

*Note:* Continuous variables are displayed as mean ± standard deviation, and categorical variables are indicated as frequency with percentage. Continuous variables are compared by ANOVA (normal distribution) or the Kruskal–Wallis H test (skewed distribution), and categorical data are compared by *χ* ^2^ test.

Abbreviations: BMI, body mass index; DBP, diastolic blood pressure; HC, hip circumference; MET‐min/week, metabolic equivalent value—minutes per week; NS, not significant; SBP, systolic blood pressure; WC, waist circumference; WHtR, waist‐to‐height ratio; WHR, waist‐to‐hip ratio.

Table 2 shows a significant increase in the percentage of total caloric intake supplied by carbohydrates, polyunsaturated fatty acids (PUFA) and fibre, across provegetarian diet index categories (*p* < 0.05). By contrast, the proportions of energy derived from protein and saturated fatty acids (SFA) and cholesterol intake generally decreased across higher score categories (*p* < 0.05). Among the micronutrients examined, vitamin C and *β*‐carotene generally increased across higher score categories, although this pattern was not maintained in the highest‐scoring category.

**Table 2:** Nutrient intakes of the study population as stratified by adherence to provegetarian dietary index categories (*N* = 205).

	Provegetarian diet index					*p*
	Category 1	Category 2	Category 3	Category 4	Category 5	
	≤ 29 (*very low*)	30–34 (*low*)	35–39 (*moderate*)	40–44 (*high*)	≥ 45 (*very high*)	
Number	18	62	75	40	10	—
Energy (kcal/day)	2350.3 ± 546.7	2198.7 ± 595.7	2296.5 ± 563.1	2156.9 ± 590.7	2599.3 ± 631.8	NS
% CHO (g/day)	42.0 ± 3.5	43.4 ± 4.5	43.3 ± 4.1	44.9 ± 4.0∗†‡	45.8 ± 4.6∗	<0.05
% protein (g/d)	18.1 ± 1.8	17.3 ± 1.5	17.1 ± 1.7∗	16.2 ± 1.2∗†‡	15.7 ± 2.3∗†	<0.0001
% total fat (g/day)	39.9 ± 2.7	39.3 ± 4.6	39.6 ± 4.0	38.8 ± 4.2	38.4 ± 4.8	NS
% SFA (g/day)	15.5 ± 1.5	14.4 ± 1.9∗	14.2 ± 1.9∗	12.9 ± 1.8∗†‡	12.6 ± 1.2∗†‡	<0.0001
% MUFA (g/day)	15.2 ± 1.4	14.8 ± 2.1	14.7 ± 1.9	14.4 ± 1.8	13.5 ± 1.6	NS
% PUFA (g/day)	9.33 ± 1.2	9.79 ± 2.1	10.5 ± 1.9∗†	11.1 ± 2.2∗†	12.0 ± 2.9∗†	<0.01
Fibre (g/day)	17.9 ± 4.5	19.1 ± 5.2	20.7 ± 5.9∗	23.5 ± 5.9∗†‡	22.6 ± 2.9∗†	<0.01
Cholesterol (mg/day)	471.3 ± 191.5	387.3 ± 120.9	352.3 ± 124.9∗	323.4 ± 108.7∗†	259.9 ± 90.4∗†	<0.0001
Vitamin C (mg/day)	44.2 ± 20.6	47.6 ± 19.1	54.3 ± 2.5∗	59.6 ± 23.9∗†	48.4 ± 6.9	<0.05
Vitamin E (mg/day)	10.26 ± 4.0	10.9 ± 4.6	10.9 ± 4.2	11.8 ± 3.4	10.9 ± 1.9	NS
Vitamin A (*μ*g/day)	2184.5 ± 2054.9	2109.2 ± 1856.8	1777.8 ± 1481.8	1661.1 ± 1430.0	1044.7 ± 560.1	NS
*β*‐carotene (*μ*g/day)	4353.9 ± 1662.1	4794.5 ± 2263.5	5243.7 ± 2532.1	6183.6 ± 3071.3∗†	5589.6 ± 1315.2∗	<0.05
Folate (*μ*g/day)	355.0 ± 110.8	369.4 ± 114.6	386.4 ± 118.5	425.6 ± 113.6	410.5 ± 95.8	NS
Vitamin B12 (*μ*g/day)	166.9 ± 64.2	166.0 ± 66.9	161.0 ± 64.0	162.4 ± 61.6	148.5 ± 35.1	NS
Iron (mg/day)	10.7 ± 4.2	10.5 ± 2.8	10.9 ± 3.1	11.9 ± 2.9	10.9 ± 2.9	NS
Zinc (mg/day)	12.3 ± 3.6	11.7 ± 3.1	11.5 ± 3.1	11.8 ± 3.1	10.7 ± 2.7	NS
Selenium (*μ*g/day)	72.5 ± 29.6	65.5 ± 22.1	66.1 ± 26.5	64.7 ± 21.4	61.2 ± 30.8	NS
Copper (mg/day)	1.92 ± 0.9	1.88 ± 0.8	1.85 ± 0.7	1.96 ± 0.7	1.65 ± 0.4	NS

*Note:* Data are displayed as mean ± standard deviation. Continuous variables are compared by one‐way analysis of variance (ANOVA) (normal distribution), or the Kruskal–Wallis H test (skewed distribution) for continuous variables.

Abbreviations: CHO, carbohydrates; MUFA, monounsaturated fatty acids; PUFA, polyunsaturated fatty acids; SFA, saturated fatty acids.

^∗^ *p* < 0.05 against Category 1.

†*p* < 0.05 against Category 2.

‡*p* < 0.05 against Category 3.

Of all food items included in the calculation of the provegetarian diet index score, only vegetables, legumes, potatoes, nuts, meat/meat products, animal fat and dairy products showed a significant difference (*p* < 0.05) across the quintile groups as depicted in Table 3. Consumption of several plant‐based food items increased significantly across higher provegetarian diet index score categories, whereas consumption of several animal‐based foods declined.

**Table 3:** Food item intakes of the study population as stratified by adherence to provegetarian diet index categories (*N* = 205).

	Provegetarian diet index					*p*
	Category 1	Category 2	Category 3	Category 4	Category 5	
	≤ 29 (*very low*)	30–34 (*low*)	35–39 (*moderate*)	40–44 (*high*)	≥ 45 (*very high*)	
Number	18	62	75	40	10	—
Vegetables (g/day)	467.4 ± 206.1	568.9 ± 245.9	659.3 ± 292.9∗†	793.7 ± 306.4∗†	761.4 ± 260.9∗†	<0.0001
Fruits (g/day)	237.7 ± 156.1	277.4 ± 221.4	351.3 ± 272.2	376.7 ± 284.3	295.1 ± 178.2	NS
Legumes (g/day)	65.9 ± 74.6	74.9 ± 57.8	100.9 ± 80.5∗†	111.3 ± 87.2∗†	137.4 ± 145.9	<0.05
Cereals (g/day)	332.6 ± 115.8	360.2 ± 157.9	335.9 ± 129.7	382.7 ± 138.6	389.1 ± 104.4	NS
Potatoes (g/day)	10.9 ± 11.6	25.8 ± 28.9∗	27.9 ± 27.2∗	52.7 ± 50.4∗†‡	58.8 ± 51.1∗†‡	<0.0001
Nuts (g/day)	14.6 ± 12.4	19.8 ± 26.2	24.0 ± 21.3∗	31.2 ± 29.3∗†	19.7 ± 16.2	<0.05
Olive oil (g/day)	4.09 ± 4.9	5.14 ± 7.0	5.52 ± 7.1	7.06 ± 5.9	7.3 ± 6.9	NS
Meat/meat products (g/day)	331.3 ± 173.3	228.9 ± 105.1∗	201.2 ± 114.7∗	181.5 ± 94.8∗†	103.3 ± 65.1∗†‡¶	<0.0001
Animal fat (g/day)	10.7 ± 6.1	7.79 ± 8.4	5.95 ± 7.0∗	5.2 ± 5.4∗	2.53 ± 2.2∗†	<0.01
Eggs (g/day)	68.9 ± 43.9	70.5 ± 62.5	53.6 ± 50.4	50.5 ± 39.3	33.5 ± 25.7	NS
Fish/seafood (g/day)	35.5 ± 39.6	31.8 ± 33.2	28.4 ± 27.5	24.5 ± 22.9	12.9 ± 11.7	NS
Dairy products (g/day)	502.6 ± 212.2	446.8 ± 210.9	378.7 ± 238.5∗	282.3 ± 197.1∗†‡	249.5 ± 161.9∗†	<0.0001

*Note:* Data are displayed as mean ± standard deviation. Continuous variables are compared by one‐way analysis of variance (ANOVA) (normal distribution) or the Kruskal–Wallis H test (skewed distribution) for continuous variables.

^∗^ *p* < 0.05 against Category 1.

†*p* < 0.05 against Category 2.

‡*p* < 0.05 against Category 3.

¶*p* < 0.05 against Category 4.

Figure 1 illustrates a subgroup analysis based on obesity status, which revealed almost similar percentages of individuals with overall and central obesity across provegetarian diet index categories (*p* > 0.05). Notably, higher percentages of females were found to have central obesity compared with overall obesity using validated threshold values of different obesity measures.

**Figure 1:**
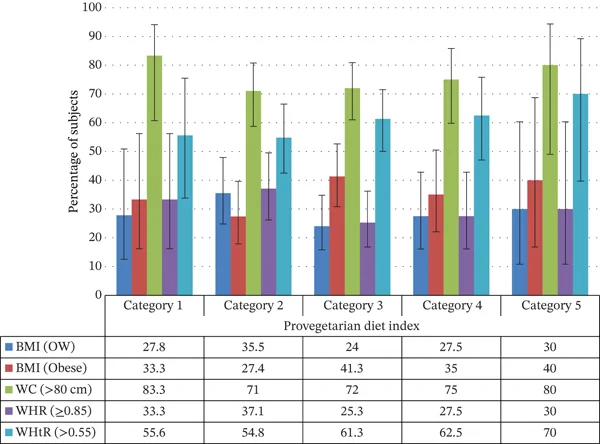
Percentage of study subjects with overweight and obesity according to anthropometric measures, as classified by adherence to provegetarian diet index categories (*N* = 205). Error bars represent 95% confidence intervals (CIs) for the corresponding percentages. BMI, body mass index; OW, overweight; WC, waist circumference; WHtR, waist‐to‐height ratio; WHR, waist‐to‐hip ratio.

Cross‐classification of provegetarian diet index categories against WHR categories above and below 0.8, a stricter threshold value of central obesity, classifies more women with central obesity in the second and third categories of the provegetarian diet index in comparison with the other groups (*p* < 0.05). This distribution was non‐monotonic. Additionally, the continuous value of provegetarian diet index is inversely correlated with WHR value (*r* = −0.144, *p* < 0.05) as depicted in Figure 2.

**Figure 2:**
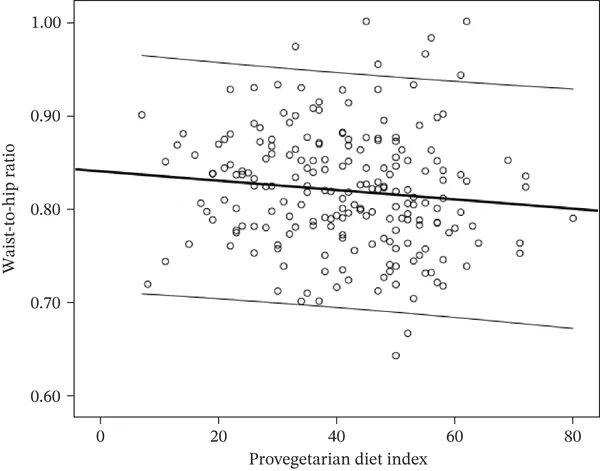
A scatter plot demonstrating correlations (*r* = −0.144, *p* < 0.05) between provegetarian diet index and waist‐to‐hip ratio among the study population (*N* = 205). The bold central line represents the fitted linear regression line, and the surrounding band represents the 95% confidence interval for the fitted mean response.

As shown in Table 4, multivariable linear regression analyses showed no significant associations between provegetarian diet index score and BMI, WC, WHR or WHtR as continuous outcomes (all *p* > 0.05). Moreover, in multivariable logistic regression, higher provegetarian diet index score was associated with lower odds of central obesity defined by WHR ≥ 0.85 (adjusted odds ratio (aOR) per one‐point increase = 0.972; 95% CI: 0.947–0.997; *p* = 0.031). No significant associations were observed for central obesity defined by WC > 80 cm or WHtR > 0.55, with complete estimates presented in Table 4.

**Table 4:** Multivariable associations between provegetarian diet index and measures of overall and central obesity.

Outcome	*N*/events†	Estimate	95% CI	*p*
Continuous outcomes				
BMI (kg/m^2^)	205	*β* = 0.039	−0.130 to 0.208	0.648
WC (cm)	205	*β* = 0.057	−0.311 to 0.426	0.759
WHR	205	*β* = −0.125	−0.002 to 0.002	0.872
WHtR	205	*β* = −0.007	−0.002 to 0.003	0.863
Binary outcomes				
Central obesity (WC >80 cm)	151/205	aOR = 0.997	0.973–1.021	0.794
Central obesity (WHR ≥ 0.85)	62/205	aOR = 0.972	0.947–0.997	0.031
Central obesity (WHtR > 0.55)	122/205	aOR = 1.007	0.985–1.029	0.548

Abbreviations: BMI, body mass index; CI, confidence interval; WC, waist circumference; WHtR, waist‐to‐height ratio; WHR, waist‐to‐hip ratio.

†Event counts are reported for binary outcomes only. B represents the unstandardised regression coefficient, corresponding to the change in the continuous outcome per one‐point increase in the provegetarian diet index. aOR represents the adjusted odds ratio per one‐point increase in the index. Models were adjusted for age, marital status, parity, occupation, monthly income, physical activity level, presence of polycystic ovary syndrome and total energy intake.

## 4. Discussion

The present study examined whether higher provegetarian diet index scores, reflecting a greater relative preference for plant‐ over animal‐derived foods, were associated with general and central adiposity among non‐pregnant Saudi women of reproductive age. Dietary differences across index score categories indicated that the index captured variation in plant‐ and animal‐food consumption. However, associations with adiposity were weak and inconsistent. The continuous index score was weakly inversely correlated with WHR (*r* = −0.144, *p* < 0.05), but multivariable analyses showed no significant associations with BMI, WC, WHR or WHtR as continuous outcomes. Higher index scores were associated with modestly lower odds of WHR‐defined central obesity (aOR = 0.972; 95% CI: 0.947–0.997; *p* = 0.031), but this finding was not consistently supported by other adiposity measures or by a monotonic pattern across index score categories. The WHR finding should therefore be considered exploratory and interpreted cautiously.

Dietary differences across provegetarian diet index score categories were generally consistent with the construction of the index [11]. Participants in higher scoring categories consumed greater amounts of several plant‐derived foods and lower amounts of several animal‐derived foods (Table 3). They also generally obtained a greater proportion of energy from PUFA and carbohydrate and consumed more fibre, vitamin C and *β*‐carotene, while obtaining less energy from protein and SFA (Table 2). The irregular pattern of fruit and nut intake in the highest‐scoring category may partly reflect its small sample size (*n* = 10). Importantly, these dietary differences were not accompanied by consistent differences in adiposity and therefore should not be interpreted as evidence that higher provegetarian diet index scores were associated with lower obesity risk. Although prospective studies have reported associations between greater adherence to plant‐based dietary patterns and lower long‐term weight gain or adiposity [20, 21], the present cross‐sectional findings provide limited support for such an association.

The absence of a clear pattern across adiposity measures is an important finding. Figure 1 shows a high prevalence of overall and central obesity, but prevalence did not decline monotonically across increasing provegetarian diet index score categories. Similarly, mean WHR did not show a consistent downward pattern across categories. Although the continuous index score was weakly inversely correlated with WHR, the association with continuous WHR was not significant after multivariable adjustment. The significant adjusted association with WHR‐defined central obesity therefore represents an isolated finding rather than evidence of a consistent dose–response relationship.

One possible explanation for the limited association with adiposity is that the provegetarian diet index primarily reflects the relative consumption of plant‐versus‐animal‐derived foods and does not explicitly distinguish foods according to their nutritional quality [22]. Plant‐derived foods differ considerably in their nutritional and metabolic characteristics; for example, refined cereals and potatoes may have different cardiometabolic associations from fruits, vegetables, legumes, nuts and whole grains [23–25]. Thus, higher index scores do not necessarily indicate an overall healthier plant‐based dietary pattern [26]. This limitation of the index may partly explain why the observed differences in dietary composition were not accompanied by consistent differences in adiposity.

Obesity is also multifactorial and reflects interactions among dietary, behavioural, metabolic and environmental determinants [27–29]. Participants with higher provegetarian diet index scores tended to report greater physical activity, although differences across score categories were not statistically significant (Table 1). Consequently, the modest association with WHR‐defined central obesity should not be attributed independently to the dietary pattern. Moreover, central adiposity may be characterised differently depending on the anthropometric measure and threshold used [30, 31]. In the present study, cross‐classification using WHR ≥ 0.80 showed a non‐monotonic distribution, with central obesity clustering in the second and third index score categories. Taken together with the null‐adjusted findings for continuous adiposity measures and the absence of consistent category‐based trends, the findings provide limited evidence for an association between provegetarian diet index scores and adiposity.

This study has several strengths. The use of a standardised provegetarian diet index enabled assessment of an overall dietary pattern rather than individual foods or nutrients, and evaluation of multiple anthropometric indicators provided complementary measures of general and central adiposity. Adjustment for relevant potential confounders further strengthened the analyses. Several limitations should nevertheless be noted. The cross‐sectional design precludes establishing temporality or causality, and residual confounding may persist. Although systematic sampling was used, pre‐enrolment refusals were not systematically recorded; therefore, participation rates and potential selection bias could not be fully assessed. Dietary assessment is susceptible to measurement error, recall bias, and reporting bias, potentially resulting in misclassification of dietary intake and consequently, provegetarian diet index scores. Furthermore, the index does not distinguish plant‐derived foods according to nutritional quality. BMI, WC, WHR and WHtR are indirect measures of adiposity and cannot distinguish fat from lean mass. The unequal distribution across index score categories, particularly the small highest‐scoring group, may have reduced the precision of subgroup estimates. Given the multiple adiposity outcomes examined and the isolated statistically significant WHR‐defined finding, Type I error cannot be excluded. The WHR association should therefore be considered exploratory and hypothesis generating.

Overall, provegetarian diet index scores were not consistently associated with measures of general or central adiposity among Saudi women of reproductive age. Although higher scores were associated with modestly lower odds of WHR‐defined central obesity, this isolated finding was not supported by consistent associations with continuous adiposity measures or monotonic patterns across score categories and should therefore be considered exploratory. Prospective studies are needed to determine whether this association is reproducible.

## Author Contributions

E.M.A. designed the research; N.M.A.M. and A.J.K. provided cases; S.A.A. conducted the subject recruitments and data collection; E.M.A. analysed the data and wrote the paper and had primary responsibility for the final content.

## Funding

This study is supported by King Abdulaziz City for Science and Technology (10.13039/501100004919; 30–37).

## Disclosure

All authors read and approved the final manuscript.

## Conflicts of Interest

The authors declare no conflicts of interest.

## Supporting information


**Supporting Information** Additional supporting information can be found online in the Supporting Information section. STROBE statement—checklist of items that should be included in reports of cross‐sectional studies.

## Data Availability

The data that support the findings of this study are available on request from the corresponding author. The data are not publicly available due to privacy or ethical restrictions.
